# Immunological and Regenerative Aspects of Hepatic Mast Cells in Liver Allograft Rejection and Tolerance

**DOI:** 10.1371/journal.pone.0037202

**Published:** 2012-05-15

**Authors:** Toshiaki Nakano, Chia-Yun Lai, Shigeru Goto, Li-Wen Hsu, Seiji Kawamoto, Kazuhisa Ono, Kuang-Den Chen, Chih-Che Lin, King-Wah Chiu, Chih-Chi Wang, Yu-Fan Cheng, Chao-Long Chen

**Affiliations:** 1 Graduate Institute of Clinical Medical Sciences, Chang Gung University College of Medicine, Niao-Sung, Taiwan; 2 Liver Transplantation Program, Kaohsiung Chang Gung Memorial Hospital, Chang Gung University College of Medicine, Niao-Sung, Kaohsiung, Taiwan; 3 Division of Transplant Immunology, Center for Translational Research in Biomedical Sciences, Kaohsiung Chang Gung Memorial Hospital, Niao-Sung, Kaohsiung, Taiwan; 4 Department of Molecular Biotechnology, Graduate School of Advanced Sciences of Matter, Hiroshima University, Kagamiyama, Higashi-Hiroshima, Japan; 5 Department of Veterinary Medicine, National Pingtung University of Science and Technology, Neipu, Pingtung, Taiwan; 6 Iwao Hospital, Kawakami, Yufu, Oita, Japan; Universidade de Sao Paulo, Brazil

## Abstract

The precise roles of mast cells in liver allograft rejection and tolerance are still unknown. This study aimed to explore the roles of mast cells in immune regulation and liver regeneration for tolerance induction by using rat models of orthotopic liver transplantation (OLT). Stem cell factor (SCF) and its receptor c-Kit, which are critical to the migration and development of not only stem cells but also mast cells, significantly increased in the tolerogenic livers as compared with rejected livers. The significant elevation of mast cell tryptase, high-affinity IgE receptor, and histamine suggested the activation of mast cells in liver allografts at the tolerogenic phase after OLT. Immunohistochemical analysis using confocal microscope clearly showed colocalization of mast cells, Foxp3^+^ Tregs, γδ T cells, and recipient-derived hepatic progenitor cells with higher expression of SCF, IL-9, IL-10, TGF-β1, and IL-17 related to immunoregulation and liver regeneration in the donor grafts of a tolerogenic OLT model. Cross-talk among mast cells and other cells was evaluated by *in vitro* studies demonstrating that syngeneic bone marrow−derived mast cells (BMMCs) co-cultured with naïve splenocytes or primary hepatocytes significantly increased the population of splenic γδ T cells by mitogen stimulation or by mast cell degranulation, and also significantly induced the hepatocyte proliferation, respectively. Our results suggested that mast cells in the donor grafts may play important roles in the induction/maintenance of immune tolerance and liver regeneration resulting in the replacement of hepatic cells from donor to recipient.

## Introduction

The first successful orthotopic liver transplantation (OLT) in humans was performed in 1963 [1], and OLT has now become a therapeutic approach for end-stage liver disease (e.g., liver cirrhosis, hepatocellular carcinoma, biliary atresia, neonatal hepatitis). For the prevention of allograft rejection, immunosuppressants such as tacrolimus (FK506) and cyclosporine have contributed to the field of organ transplantation and are an effective therapeutic modality [2]. Pharmacological roles of immunosuppressants in the tolerance process are not yet clarified, but it has been suggested the adverse effect of immunosuppressants on the development of Foxp3^+^ regulatory T cells (Tregs) [3], [4], [5]. Furthermore, Danecke et al. demonstrated the interference of cyclosporine with tolerance induction *in vivo* [6]. To date, random trials to withdraw immunosuppressive drugs from long-time surviving patients who not had a rejection episode are currently being performed. However, these random trials, which lack unified standard to evaluate the degree of rejection, have not precisely determined the timing when immunosuppressive therapy should be reduced or terminated. As a result, some recipients would have the good fortune to achieve operational tolerance with increased number of Tregs, including Foxp3^+^ T cells and γδ T cells in peripheral blood after liver transplantation [7], [8], but other patients would have a risk of rejection again. Therefore, complete understanding of humoral and cellular events in the rejection and tolerance process is crucial to safety withdraw immunosuppressive drugs, which will contribute to alleviate the physical, mental, and financial anguish related to liver transplantation therapy.

In general, organ allograft rejection can be defined as an immunological reaction in response to the presence of a foreign tissue or organ, which may potentially result in graft dysfunction and failure [9]. Liver allograft rejection has been divided into hyperacute (humoral), acute (cellular), and chronic (ductopenic and/or arteriopathic) processes [10]. In recent years, many studies have suggested that hepatic mast cells are involved in chronic rejection [11], hepatic fibrogenesis [12], [13], [14], [15], and cholestatic liver diseases [13], [16]. Although mast cells are seen in normal liver surrounding the vessels and the bile ducts in the portal tracts, and to a much lesser extent, in the hepatic parenchyma [13], hepatic mast cells infiltrating portal tracts and surrounding damaged bile ducts have been focused as important effector cells in the pathogenesis of chronic rejection [11]. Immunologically, mast cells are important effector cells in a broad range of immune response [12]. Immediate hypersensitivity and host defense mechanisms against parasites are closely related to mast cell function [17]. Furthermore, mast cells have been implicated in fibrosis related to the rejection process of other organ transplantation including renal [18], [19], [20], lung [21], and heart transplantation [22], [23].

On the other hand, findings in skin transplantation by Lu et al. [24] have provided substantial evidence for the role of mast cells in Treg−mediated immunoregulatory activities by demonstrating that mast cells are crucial for allograft tolerance through the inability to induce tolerance in mast-cell-deficient mice. In addition, Boerma et al. reported that the absence of mast cells is associated with significantly reduced cardiac allograft survival after heterotopic heart transplantation in rats [25]. However, until recently, little attention has been given to the study of hepatic mast cells in terms of Treg−mediated immune tolerance such as Foxp3^+^ T cells and γδ T cells since the precise roles of hepatic mast cells in tolerogenic livers have not been elucidated.

In the present study, we characterize hepatic mast cells and related factors, such as stem cell factor (SCF), c-Kit, high-affinity IgE receptor (FcεRIα) and mast cell−specific enzymes, in liver allografts at the rejection and tolerogenic phases and discuss the immunological aspects of hepatic mast cells on liver allograft rejection and tolerance by using rat models of OLT in the rejecting and tolerogenic combinations. We also discuss the significance of migration and differentiation of bone marrow−derived stem cells into hepatic stem/progenitor cells in tolerogenic livers after OLT.

## Results

### Liver histology in rejected and tolerant livers

**Figure 1:**
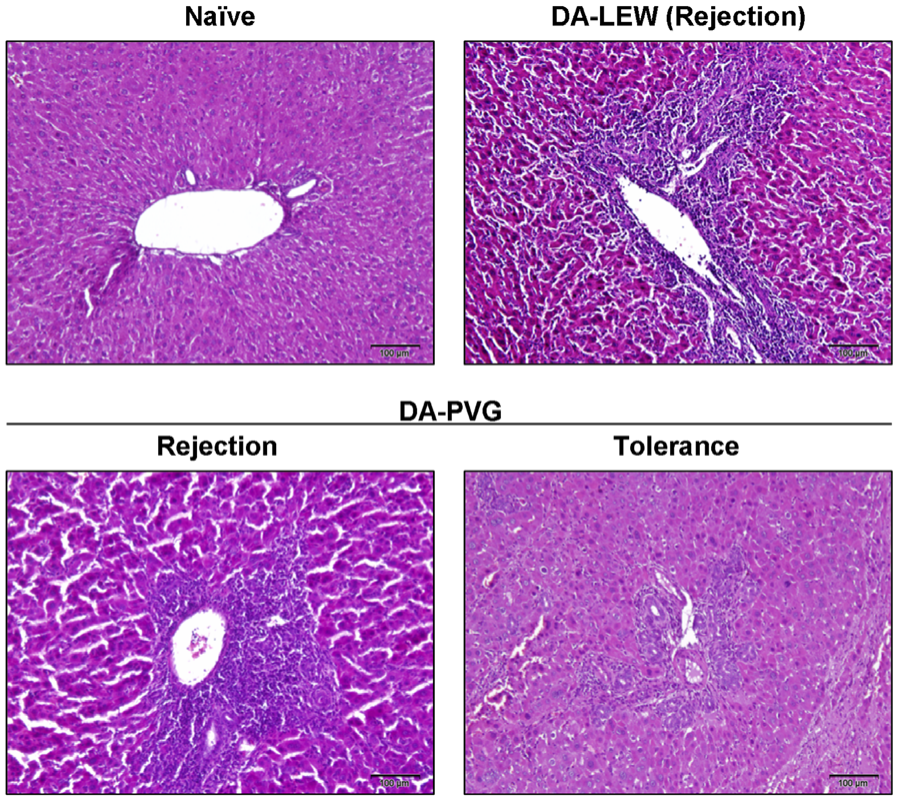
Histological evaluation of rejection after OLT. Cryosections of naïve and OLT livers at rejection (day 7) and tolerogenic phase (>60 days) were stained with hematoxylin and eosin. Data are representative of six individual liver sections (10× magnification, bar = 100 µm).

Immunology in liver transplantation is unique and depends on combination of rat strains used for donors and recipients. In the DA donor livers into PVG recipients, allograft rejection is spontaneously overcome after OLT, resulting in a state of long-lasting and donor-specific tolerance without pharmacological immunosuppression although PVG recipients acutely reject skin, heart, and renal grafts from DA rats [26]. Interestingly, PVG recipients bearing DA livers could accept skin, heart, and kidney from the DA donor rats but rejected them from the third party strains of rats [27], [28]. This donor-specific tolerance could be transferred to naïve animals by serum transfusion obtained from the recipient PVG rats >60 days after OLT [29]. In contrast, recipient LEW rats usually reject a donor DA rat liver within 14 days after OLT [30]. Liver histology demonstrated the massive infiltration of immune cells and the damage to hepatic parenchyma at the rejection phase (day 7) after OLT both in DA-PVG and DA-LEW combinations, while tolerogenic livers at >60 days after OLT showed less inflammation (Fig. 1).

### Significance of SCF in liver allograft tolerance

SCF is a growth factor that is important for the survival, proliferation and differentiation of hematopoietic stem cells and other hematopoietic progenitor cells, including mast cells [31]. To explore the role of hepatic mast cells in liver allograft rejection and tolerance, we checked the hepatic levels of SCF. As shown in Table 1, the hepatic level of SCF significantly increased at the tolerogenic phase after OLT compared with naïve and rejected livers, suggesting the presence of c-Kit^+^ hematopoietic stem/progenitor cells and mast cells in tolerogenic livers.

**Table 1:** Relative gene expression of SCF, c-Kit, Tpsab1, FcεRIα, and TNF-α.

mRNA	Naïve DA	DA-PVG		DA-LEW
		Rejection	Tolerance	Rejection
SCF	0.882±0.229	2.07±1.66	3.47±1.45*	1.07±0.267
c-Kit	0.958±0.203	1.91±0.400**	1.64±0.573**	0.857±0.368
Tpsab1	0.635±0.373	0.581±0.500	4.94±1.99**	0.773±0.399
FcεRIα	0.989±0.226	4.20±2.49*	6.19±3.85*	2.70±1.01*
TNF-α	1.76±1.60	12.7±7.24**	3.47±2.98	14.7±4.71**

*,** Significantly different compared with naïve DA livers (n = 9) (*P*<0.05 and 0.01, respectively).

### Activation of hepatic mast cells in liver allografts at the tolerogenic phase after OLT

We next evaluated hepatic mast cell activation by quantitative real-time PCR analysis. As shown in Table 1, hepatic level of c-Kit significantly increased both at rejection and tolerogenic phases in a rat tolerogenic OLT model. On the other hand, we observed significant elevation of mast cell tryptase (Tpsab1) and high-affinity IgE receptor (FcεRIα) at the tolerogenic phase after OLT, suggesting the activation of hepatic mast cells in liver allografts at the tolerogenic phase after OLT. The lower expression of TNF-α in liver allografts at the tolerogenic phase suggested that the activation of hepatic mast cells might not be associated with chronic rejection.

### Induction of γδ T cells and Foxp3^+^ regulatory T cells after OLT

To explore the roles of Tregs in liver allograft tolerogenicity, we next evaluated the hepatic levels of Foxp3, TCR γ chain, and the inhibitory cytokine IL-10 at both the rejection and tolerogenic phases after OLT. As shown in Table 2, the early elevation of Foxp3, TCR γ chain, and IL-10 suggested the involvement of Tregs in overcoming rejection and the subsequent tolerance induction.

**Table 2:** Relative gene expression of Foxp3, TCR γ chain, and IL-10.

mRNA	Naïve DA	DA-PVG		DA-LEW
		Rejection	Tolerance	Rejection
Foxp3	2.85±2.88	111±44.9**	63.2±39.7*	56.4±9.86**
TCR γ	1.37±0.928	16.1±8.72*	7.26±1.26**	5.25±1.66*
IL-10	4.13±4.26	65.5±24.5**	13.6±1.62*	28.9±18.2*

*,** Significantly different compared with naïve DA livers (n = 9) (*P*<0.05 and 0.01, respectively).

### Colocalization of hepatic mast cells and Tregs in tolerogenic liver allografts

**Figure 2:**
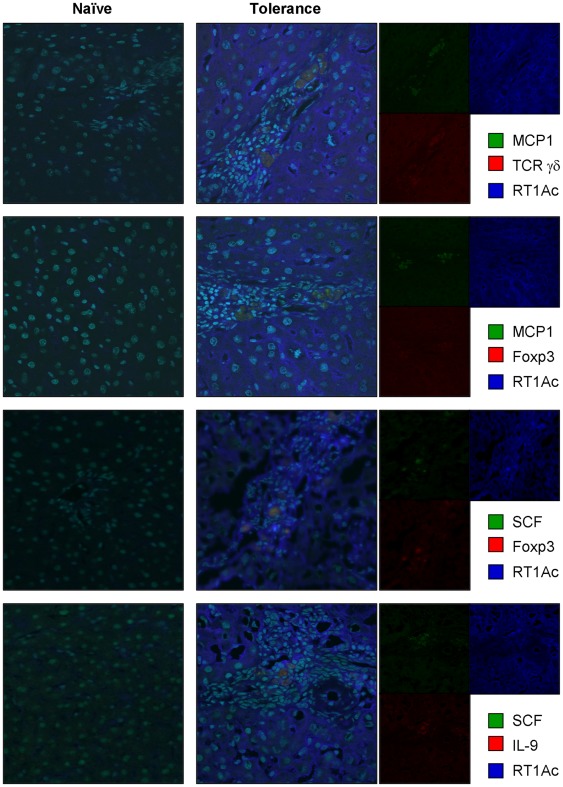
Colocalization of recipient-derived hepatic mast cells, Tregs (Foxp3^+^/TCR γδ chain^+^) and cytokines (SCF and IL-9) for mast cell activation. Cryosections of naïve and OLT livers at tolerogenic phase (>60 days) were immunoprobed with goat polyclonal Ab against MCP1, mouse monoclonal Ab against γδ TCR, Foxp3 or IL-9, rabbit polyclonal Ab against SCF, and rat polyclonal Ab against RT1Ac (specific to PVG recipients) followed by incubation with Alexa Fluor® 488−conjugated donkey anti-goat or rabbit IgG, HyLite Fluor™ 594−conjugated goat anti-mouse IgG or Alexa Fluor® 647−conjugated goat anti-rat IgG. Hoechst 33342 (specific to nucleus) was used for counterstaining. Data are representative of six individual liver sections (60× magnification). Right columns indicate the data without merging.

**Figure 3:**
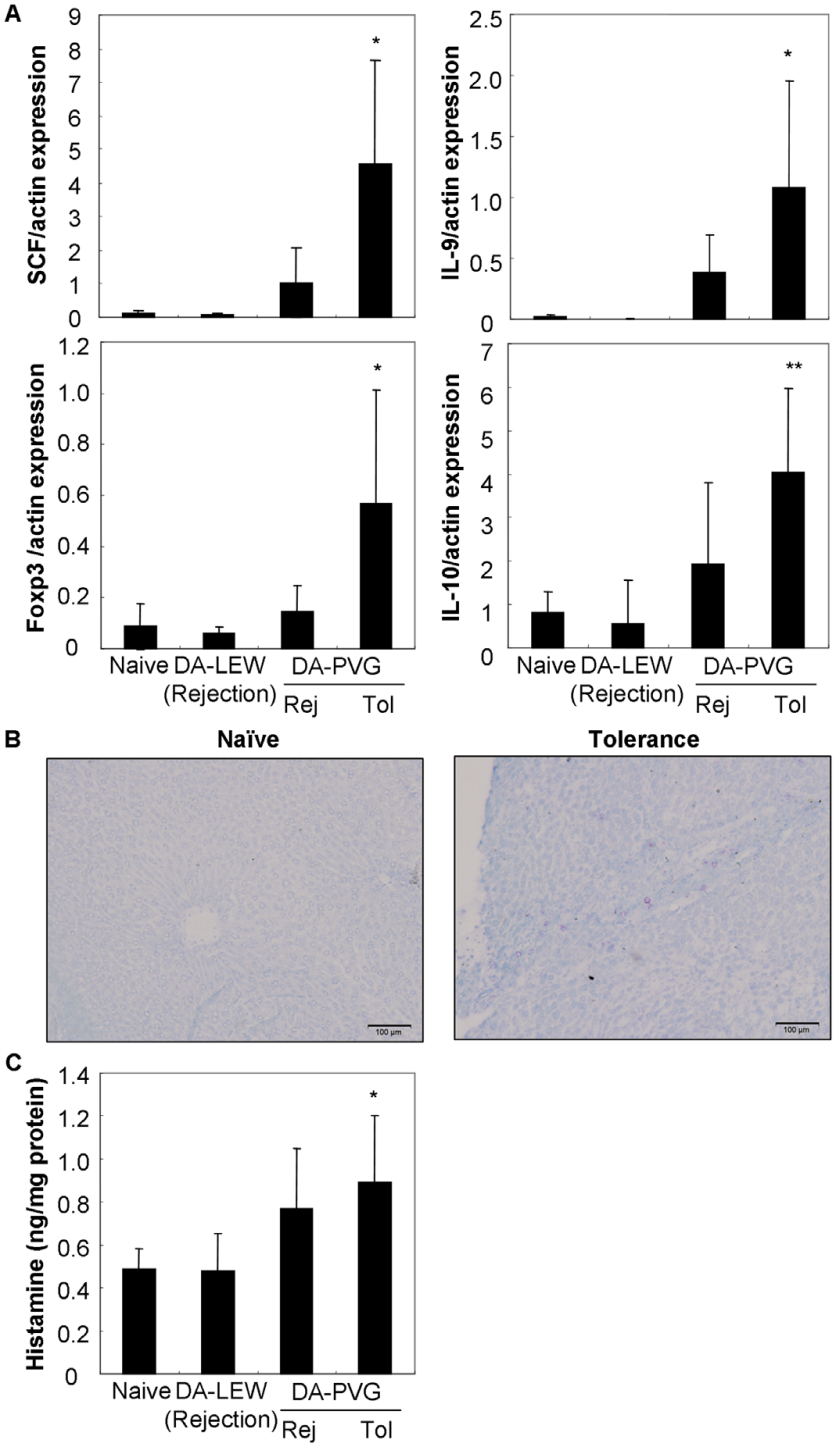
Hepatic levels of SCF and IL-9 are correlated with Foxp3/IL-10 expression and mast cell activation in tolerogenic liver allografts. (A) Protein levels of SCF, IL-9, Foxp3, and IL-10 in naïve and OLT livers at rejection (Rej; day 7) and tolerogenic phase (Tol; >60 days) were semi-quantified by Western blot. The data were normalized to actin expression. Results are expressed as the mean of six individuals ± SD. *, ** Significantly different compared with naïve livers (*P*<0.05 and 0.01, respectively). (B) The granulated mast cells were histochemically stained with toluidine blue. Granules of mast cells were metachromatically stained red-purple. Data are representative of six individual liver sections (10× magnification, bar = 100 µm). (C) Hepatic level of histamine was quantified using a specific ELISA kit and normalized to the total protein (1 mg) of liver extracts. Results are expressed as the mean of six individuals ± SD. * Significantly different compared with naïve livers (*P*<0.05).

Lu et al. have provided evidence that CD4^+^Foxp3^+^ Tregs seem to produce IL-9 and, through the production of IL-9, may mediate the recruitment and activities of mast cells *in vivo* [24]. To characterize the association between hepatic mast cells and Tregs in liver allograft tolerance, immunohistochemical analysis was performed. As shown in Fig. 2, we observed the colocalization of hepatic mast cells (mast cell protease 1 [MCP1]-producing cells) and Tregs (Foxp3^+^ cells and γδ T cells) in liver allografts at the tolerogenic phase after OLT. Furthermore, we observed the production of SCF by Foxp3^+^ Tregs. SCF and IL-9 were colocalized in the tolerogenic liver allografts, suggesting Foxp3^+^ Tregs produced these cytokines to activate hepatic mast cells. Protein levels of SCF and IL-9 in liver allografts were also correlated with the activation of Foxp3 and IL-10 production (Fig. 3A), resulting in hyperplasia and the activation of hepatic mast cells as assessed by toluidine blue staining (Fig. 3B) and histamine production (Fig. 3C). In addition, we observed the replacement of both hepatocytes and nonparenchymal cells from the donor MHC haplotype (RT1Aa) to the recipient haplotype (RT1Ac) (Fig. 2).

### TGF-β1/IL-17−producing mast cells and γδ T cells in tolerogenic liver allografts

**Figure 4:**
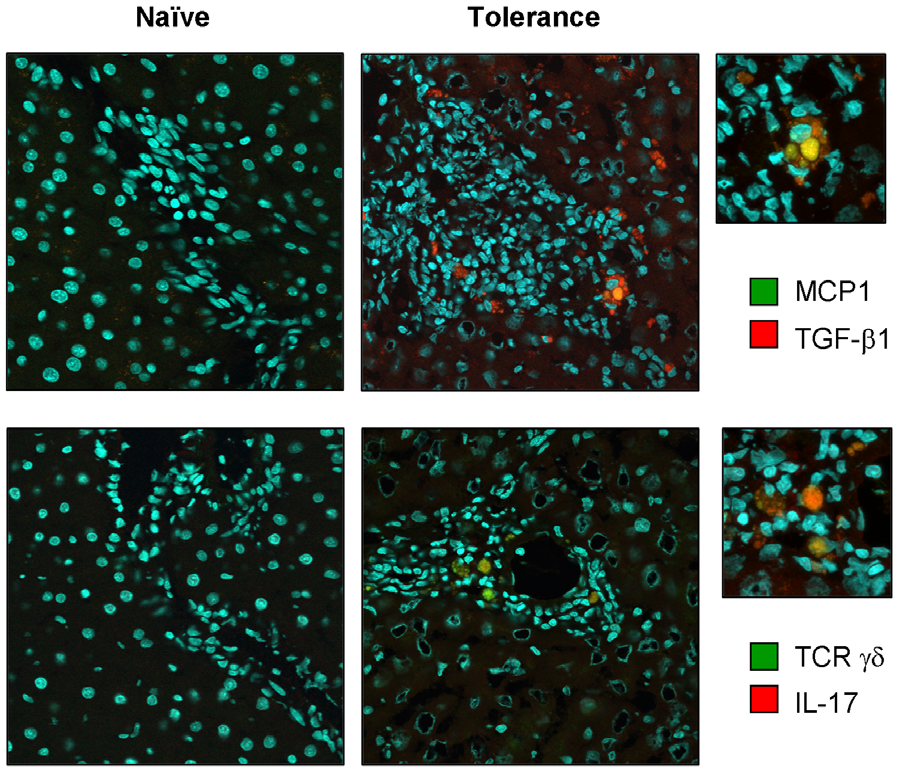
TGF-β1−producing mast cells and IL-17−producing γδ T cells in tolerogenic liver allografts. Cryosections of naïve and OLT livers at tolerogenic phase (>60 days) were immunoprobed with goat polyclonal Ab against MCP1, mouse monoclonal Ab against γδ TCR, and rabbit polyclonal Ab against TGF-β1 or IL-17 followed by incubation with Alexa Fluor® 488−conjugated donkey anti-goat or mouse IgG or HyLite Fluor™ 594−conjugated goat anti-rabbit IgG. Hoechst 33342 (specific to nucleus) was used for counterstaining. Data are representative of six individual liver sections (60× magnification). A higher magnification (210×) was used to show the colocalization of TGF-β1/MCP1 and IL-17/TCR γδ chain in tolerogenic liver allografts (upper right column).

Mast cells produce TGF-β1 to regulate Treg−mediated immune tolerance *in vitro* [32]. On the other hand, γδ T cells are innate sources of IL-17, a potent proinflammatory cytokine mediating bacterial clearance as well as autoimmunity [33], [34], [35]. To further explore the cross-talk among hepatic mast cells, Foxp3^+^ Tregs, and γδ T cells in liver allograft tolerogenicity, we performed immunohistochemical analysis. As shown in Fig. 4, we observed the production of TGF-β1 and IL-17 by hepatic mast cells and γδ T cells, respectively.

### Induction of γδ T cells by mast cells co-cultured with splenocytes

**Figure 5:**
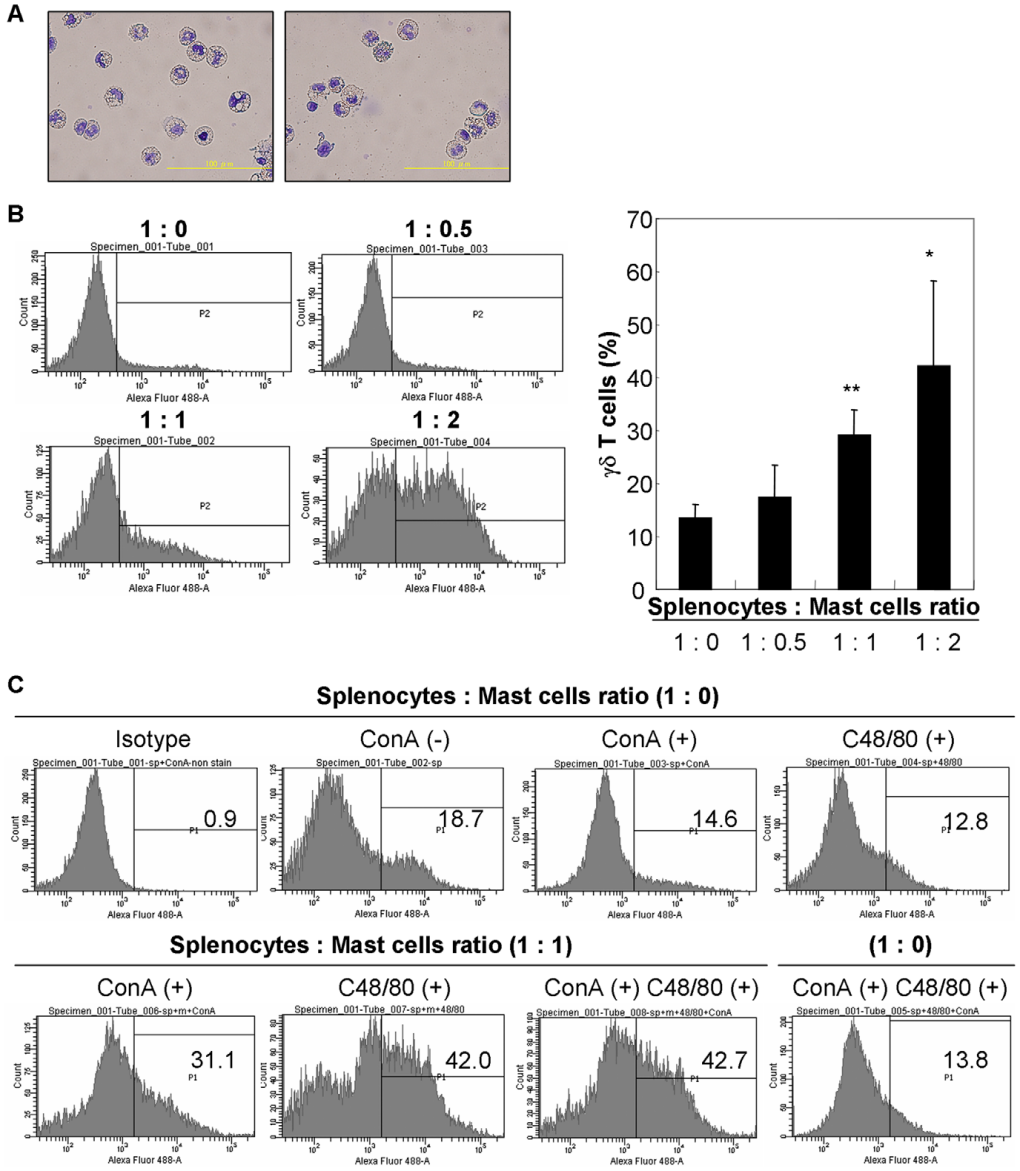
Induction of splenic γδ T cells by mitogen stimulation and co-cultured with BMMCs or pharmacological induction of mast cell degranulation. (A) The purity of BMMCs was evaluated by Toluidine blue staining. Data are representative of three independent experiments (40× magnification, bar = 100 µm). (B) Splenocytes (2.5×10^6^ cells) were co-cultured with syngeneic BMMCs (0, 1.25, 2.5 and 5×10^6^ cells) and stimulated with Concanavalin A (2.5 µg/ml) for 72 hrs. Histograms show the expression level of TCR γδ chain on splenic cells. Data are representative of three independent experiments. *, ** Significantly different compared with control (splenocytes∶mast cell ratio = 1∶0) (*P*<0.05 and 0.01, respectively). (C) Splenocytes (2.5×10^6^ cells) were co-cultured with syngeneic BMMCs (0 and 2.5×10^6^ cells) and stimulated with compound 48/80 (C48/80, 10 µg/ml) with/without Concanavalin A (2.5 µg/ml) for 72 hrs. Histograms show the expression level of TCR γδ chain on splenic cells. C48/80 has no effect on the generation of γδ T cells without BMMCs. Data are representative of three independent experiments.

Zhang et al. recently demonstrated the induction of CD4^+^CD25^+^Foxp3^+^ Tregs when bone marrow-derived mast cells (BMMCs) were co-cultured with T cells *in vitro* [32]. However, little is known about the cross-talk between mast cells and γδ T cells. To investigate whether BMMCs can induce splenic γδ T cells *in vitro*, splenocytes were co-cultured with syngeneic BMMCs together with mitogen stimulation. As shown in Fig. 5B, the percentage of γδ T cells significantly increased in a cell ratio-dependent manner. To evaluate the role of humoral factors secreted from the mast cells, compound 48/80, which is known as a histamine-liberating substance [36], was added in the culture medium. Pharmacological induction of mast cell degranulation by compound 48/80 strongly induced the γδ T cells generation, suggesting the involvement of mast cell mediators such as histamine for activation of γδ T cells (Fig. 5C).

### Significance of migration and transdifferentiation of bone marrow−derived stem cells into hepatocytes and non-parenchymal cells in tolerogenic livers

**Figure 6:**
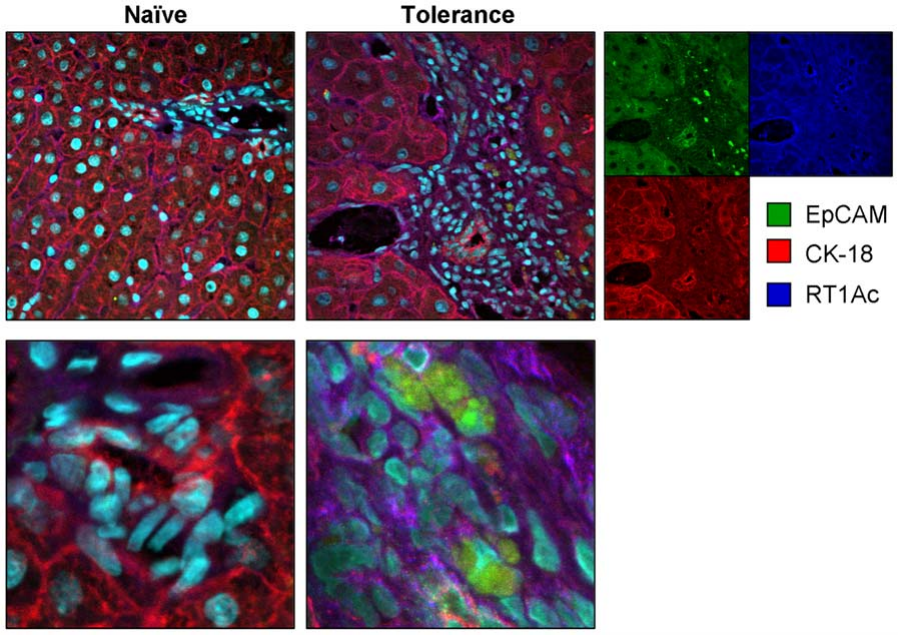
Existence of recipient-derived hepatic cells in tolerogenic liver allografts. Cryosections of naïve and OLT livers at tolerogenic phase (>60 days) were immunoprobed with rabbit monoclonal Ab against EpCAM, mouse monoclonal Ab against CK-18, and rat polyclonal Ab against RT1Ac (specific to PVG recipients) followed by incubation with Alexa Fluor® 488−conjugated donkey anti-rabbit IgG, HyLite Fluor™ 594−conjugated goat anti-mouse IgG or Alexa Fluor® 647−conjugated goat anti-rat IgG. Hoechst 33342 (specific to nucleus) was used for counterstaining. (Upper columns) Data are representative of six individual liver sections (60× magnification). Right columns indicate the data without merging. (Bottom columns) A higher magnification (220×) was used to show the colocalization of EpCAM, CK-18 and RT1Ac in tolerogenic liver allografts.

Combining granulocyte colony-stimulating factor with a dipeptidylpeptidase-IV (DPP-IV) inhibitor can promote mobilization and migration of recipient bone marrow−derived hematopoietic stem cells into ischemically injured areas, resulting in tissue regeneration after acute myocardial infarction [37]. Hepatic progenitor cells appear in injured livers when hepatocyte proliferation is impaired. These cells can differentiate into hepatocytes and cholangiocytes [38]. To explore the migration and transdifferentiation of bone marrow−derived stem cells into hepatic cells in tolerogenic livers, we next evaluated the hepatic levels of DPP-IV and markers for hepatic progenitor cells, such as α-fetoprotein (AFP), epithelial cell adhesion molecule (EpCAM), which marks hepatocytes newly derived from hepatic stem/progenitor cells [39], and cytokeratin (CK)-18, which is known as a hepatocytic marker [40], at the rejection and tolerogenic phases after OLT. As shown in Table 3, DPP-IV level in OLT livers was significantly lower than that in naïve livers, suggesting the accessibility of bone marrow−derived stem cells to the rejected and tolerant livers. In addition to the c-Kit (Table 1), on the other hand, the hepatic level of AFP significantly increased in the DA-PVG rejected livers, suggesting the transdifferentiation of bone marrow−derived stem cells into hepatic progenitor cells at the rejection phase in a rat tolerogenic OLT model. AFP level in the tolerogenic livers was significantly lower than that in naïve livers, while EpCAM and CK-18 were significantly higher in the tolerogenic livers compared with naïve and rejected livers. We also confirmed the existence of recipient-derived EpCAM^+^/CK-18^+^ hepatic cells newly derived from the hepatic progenitor cells, suggesting the hepatic differentiation of bone marrow−derived stem cells in tolerogenic livers (Fig. 6).

**Table 3:** Relative gene expression of DPP-IV, AFP, EpCAM, and CK-18.

mRNA	Naïve DA	DA-PVG		DA-LEW
		Rejection	Tolerance	Rejection
DPP-IV	1.36±0.401	0.480±0.375**	0.610±0.256**	0.271±0.108**
AFP	0.998±0.346	5.18±1.37**	0.581±0.112*	0.688±0.826
EpCAM	1.09±0.579	1.35±1.23	4.45±2.43**	1.60±0.972
CK-18	0.934±0.233	1.22±0.833	2.44±0.874**	1.49±0.958

*,** Significantly different compared with naïve DA livers (n = 9) (*P*<0.05 and 0.01, respectively).

### Induction of liver regeneration by mast cells co-cultured with primary hepatocytes

**Figure 7:**
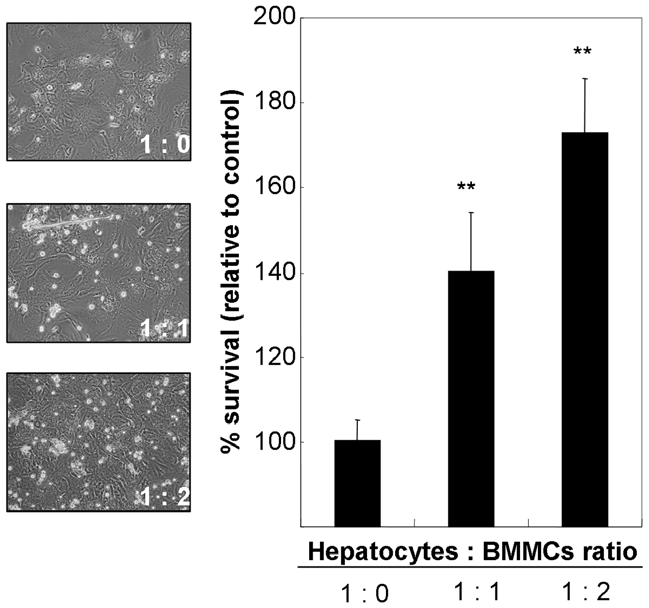
Induction of liver regeneration by BMMCs. Primary hepatocytes (2×10^5^ cells) were co-cultured with syngeneic BMMCs (0, 2 and 4×10^5^ cells) for 72 hrs. After washing twice with PBS, new culture medium was added together with MTT reagents, cultured for 4 hrs and the absorbance (595 nm) was measured after color development. Percent survival of hepatocytes relative to control (hepatocytes∶mast cell ratio = 1∶0) is represented as the mean of six independent culture wells ± SD. ** Significantly different compared with control (*P*<0.01).

To investigate the direct role of mast cells on liver regeneration, primary hepatocytes were co-cultured with syngeneic BMMCs and hepatic cell growth was evaluated by MTT assay. As shown in Fig. 7, the percent survival of hepatocytes significantly increased in a cell ratio-dependent manner.

## Discussion

In this study, we revealed the dynamics of c-Kit, mast cells, Tregs, and hepatic stem/progenitor cells in liver allograft rejection and tolerance. We confirmed the elevation of c-Kit in liver allografts at the rejection phase, particularly in a rat tolerogenic OLT model (DA-PVG). Based on the lower expression of mast cell tryptase in rejected livers, we speculate that the early induction of c-Kit may reflect the migration of endogenous hematopoietic stem/progenitor cells derived from bone marrow to repair/replace damaged tissue rather than to activated mast cells. In support of our hypothesis, we observed the massive replacement of both hepatocytes and nonparenchymal cells from the donor MHC haplotype (RT1Aa) to the recipient haplotype (RT1Ac) at the tolerogenic phase after OLT. We also confirmed the elevation of AFP in the DA-PVG rejected livers probably reflecting the induction of hepatic stem/progenitor cells in the liver allografts, resulting in the liver regeneration for replacement of donor-type hepatic cells. Replacement of donor Kupffer cells by recipient cells after OLT has been reported previously [41], [42], resulting in the mild rejection response observed in both experimental and clinical liver transplantation. In addition, transdifferentiation of hematopoietic stem cells into hepatocytes was reported in rats [43], humans [44], and mice [45]. Theise et al. reported that 4 to 43% of hepatocytes were replaced by recipient (male, Y chromosome^+^) cells after grafting of female liver allografts [44]. However, the transdifferentiation of recipient bone marrow−derived stem cells into parenchymal cells is an extremely rare event in liver, small intestine, and heart allografts [46]. Our animal model may be a good resource for understanding the transdifferentiation of endogenous stem cells in liver allograft tolerogenicity.

In addition to the induction of c-Kit^+^ cells, Foxp3^+^ Tregs, and γδ T cells dramatically migrated to liver allografts at the rejection phase after OLT. Foxp3^+^ Tregs possess immune-regulatory functions by producing the anti-inflammatory cytokines IL-10 and TGF-β1 [47], [48]. Similar to our data, Li et al. recently reported the induction of Foxp3 and IL-10 in liver allografts at the rejection phase after OLT in BN-to-LEW combinations [49]. The gene expression of Foxp3 and IL-10 in the rejected livers (day 7) was higher than those in the tolerant livers (>day 60), while the protein expression of Foxp3 and IL-10 was opposite fashion. These data suggested that Foxp3 and IL-10 were transcriptionally active but translationally inactive in the rejected livers. Time lags between transcription and translation of Foxp3 and IL-10 are predicted and may explain the different patterns of gene and protein expression. γδ T cells are innate-type lymphocytes that act as regulators of local effector immune responses and are an innate source of IL-17, resulting in the enhancement of autoimmunity [33], [34], [35]. They are abundant in the liver and are involved in anti-tumor surveillance and immune regulation [50]. However, the fundamental roles of γδ T cells in transplant rejection and tolerance have not been well documented. In this study, we demonstrated the upregulation of γδ T cells in liver allografts compared with naïve DA livers. In our previous studies, we demonstrated that the autoimmune response against nuclear antigens such as histone H1 might be an important phenomenon in overcoming rejection and in subsequent tolerance induction in a rat tolerogenic OLT model [51], [52], [53], [54]. The significance of autoimmunity against nuclear antigens such as histones and high-mobility group box 1 in immune regulation has been demonstrated [55], [56]. We speculate that the existence of anti-nuclear auto-Abs in the systemic circulation may regulate uncontrolled immune responses and that γδ T cells may regulate the balance of autoimmunity and alloimmunity. Taken together, the early induction of c-Kit, Foxp3^+^ Tregs, and γδ T cells may be indispensable for overcoming acute rejection and the subsequent tolerance induction.

**Figure 8:**
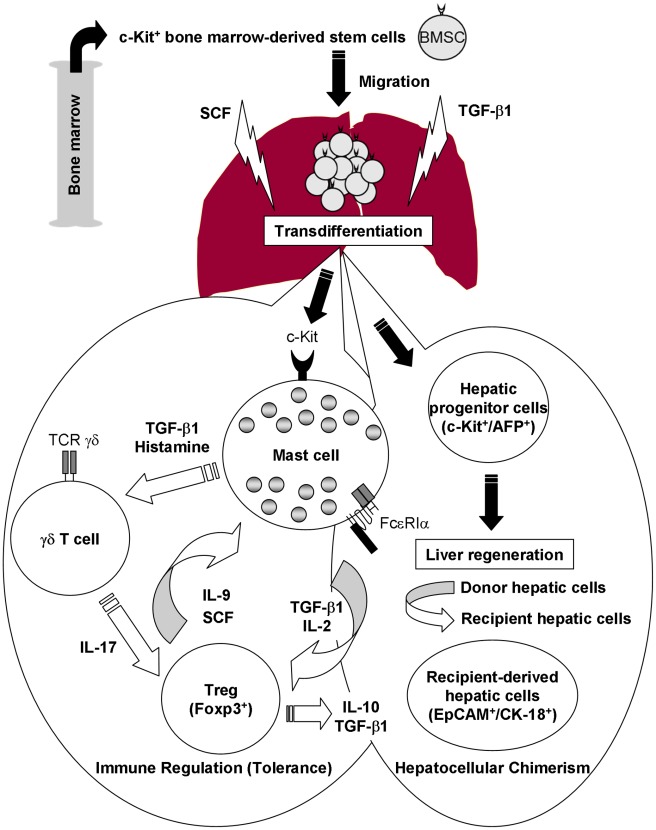
Possible association among hepatic mast cells, Foxp3^+^ Tregs and γδ T cells for immune regulation and hepatocellular chimerism. Under the guidance of SCF/c-Kit signaling, hematopoietic stem/progenitor cells migrate to the liver allografts to induce liver allograft tolerance and simultaneously induce liver regeneration for the replacement of donor with recipient hepatic cells. Foxp3^+^ Tregs may produce IL-9 and SCF to activate hepatic mast cells; in turn, activating mast cells may produce TGF-β1, and IL-2 and/or release histamine by degranulation. TGF-β1 also plays a key role in the generation of IL-17−producing γδ T cells; in turn, IL-17 indirectly attracts Foxp3^+^ Tregs, enhances their suppressor function, and induces IL-9 and SCF production by Foxp3^+^ Tregs. Production of SCF, TGF-β1, and IL-10 also may regulate liver regeneration.

In this study, we explored the cross-talk among hepatic mast cells, Foxp3^+^ Tregs, and γδ T cells in liver allograft tolerogenicity. In addition to the production of IL-9, Foxp3^+^ Tregs may produce SCF to activate hepatic mast cells; in turn, activating mast cells may produce TGF-β1. It has also been demonstrated the cross-talk between mast cells and Tregs through the production of IL-2 by mast cells which enhances the proportion of Tregs at the site of inflammation [57]. On the other hand, IL-17 indirectly attracts Foxp3^+^ Tregs, enhances their suppressor function, and induces IL-9 production by Foxp3^+^ Tregs; in turn, IL-9 strengthens the survival and protumor effects of mast cells in the tumor microenvironment [58]. TGF-β1 also plays a key role in the generation of IL-17−producing γδ T cells [35]. Our *in vitro* data also demonstrated the generation of γδ T cells by pharmacological induction of mast cell degranulation. In support of our data, it has been demonstrated the activation of γδ T cells by histamine [59]. From the regenerative point of view, SCF/c-Kit signaling, IL-10, TGF-β1, and histamine play important roles in the regulation of liver regeneration [60], [61], [62], [63]. Our *in vitro* data clearly demonstrated the induction of hepatic cell proliferation by co-culture with mast cells. Based on our present and other findings, we propose an intrinsic relationship among mast cells, Foxp3^+^ Tregs, and γδ T cells in tolerogenic livers (Fig. 8).

In summary, our data suggest that early induction of c-Kit, Foxp3^+^ Tregs, and γδ T cells may be indispensable for overcoming acute rejection and that Foxp3^+^ Tregs, γδ T cells, and hepatic mast cells may play important roles in the induction and maintenance of immune tolerance and liver regeneration by producing SCF, IL-9, IL-10, TGF-β1, IL-17, and histamine. Massive replacement of donor cells, including hepatocytes, by recipient cells may contribute to overcoming the rejection and the subsequent induction of tolerance without immunosuppressive treatment in the DA-PVG combination. Further studies, including a cell-based analysis of cytokine production, cell−cell interactions, and the effects of various cytokines and cells on liver regeneration, should be performed to improve our understanding of humoral and cellular responses in liver allograft tolerogenicity.

## Materials and Methods

### Ethics statement

Our experimental design was reviewed and approved by the Institutional Animal Care and Use Committee (approval No: 2009080401), and the Committee recognizes that the proposed animal experiment follows the Animal Protection Law by the Council of Agriculture, Executive Yuan, R.O.C. and the guideline as shown in the Guide for the Care and Use of Laboratory Animals as promulgated by the. Institute of Laboratory Animal Resources, National Research Council, USA.

### Animals

Male DA (MHC haplotype RT1^a^) and PVG (RT1^c^) rats that were 4 weeks of age were obtained from Japan SLC (Hamamatsu, Japan) and the Institute of Laboratory Animals of the Graduate School of Medicine, Kyoto University (Kyoto, Japan), respectively. Male LEW (RT1^l^) rats 4 weeks of age were obtained from the National Laboratory Animal Breeding and Research Center (Taipei, Taiwan). All animals were maintained in specific pathogen−free animal facilities with water and commercial rat food provided *ad libitum*.

### Orthotopic liver transplantation

OLT was carried out following the technique previously described by Kamada et al. [64] in the following combinations: DA to PVG (DA-PVG; natural tolerance model) and DA to LEW (DA-LEW; acute rejection model). All serum samples and liver grafts were stored at −80°C until analysis.

### RNA isolation and real-time PCR

RNA was extracted using REzol™ reagent (Promega Corporation, Madison, WI, USA) according to the manufacturer's instructions from a rat liver harvested at the rejection phase (postoperative day 7: n = 6) and at the tolerogenic phase (postoperative day 60 to 85: n = 6). Total RNA (1.2 µg) was reverse-transcribed into cDNA with ImProm-II™ Reverse Transcriptase (Promega Corporation). The rat-specific PCR primers are shown in Table 4. Quantitative PCR of GAPDH, SCF, c-Kit, mast cell tryptase (Tpsab1), FcεRIα, TNF-α, Foxp3, TCR γ chain, IL-10, DPP-IV, AFP, EpCAM, and CK-18 cDNAs was performed using a 7500 Fast Real-Time PCR System (Applied Biosystems Inc., Foster, CA, USA). The GAPDH reference gene was used to normalize the data. The 2^−Δ.CT^ value, which corresponds to the expression of each gene compared to GAPDH, and 2^−Δ.Δ.CT^, which corresponds to the expression ratio of each gene in the experimental group compared to the control, were calculated.

**Table 4:** Characteristics of primers.

mRNA	Sense primer	Antisense primer
	5′ 3′	5′ 3′
GAPDH	CCATGGAGAAGGCTGGGG	CAAAGTTGTCATGGATGACC
SCF	CAAAACTGGTGGCGAATCTT	GCCACGAGGTCATCCACTAT
c-Kit	AAGCCGAGGCCACTCACACGGGCAAAT	CCAACCAGGAAAAGTACGGCAGGATCTC
Tpsab1	ACATCTGAGTGTTGCGCTGAAGCA	CCCAACAGGTTGTGGTGTGCAGAAT
FcεRIα	CATTGTGAGTGCCACCATTC	TTCTTCCAGCTACGGCATCT
TNF-α	TGCCTCAGCCTCTTCTCATT	GCTTGGTGGTTTGCTACGAC
Foxp3	CCCAGGAAAGACAGCAACCTT	CTGCTTGGCAGTGCTTGAGAA
TCR γ	ACAGCAATACGATCCTGGACTCCCA	ACCCTTCAGGCACAGTAAGCCA
IL-10	CAGACCCACATGCTCCGAGA	CAAGGCTTGGCAACCCAAGTA
DPP-IV	TCAAGTCCTGCTCTTCCACTGCAA	AGAACTTTGCACATGGCTGCTGCT
AFP	CAGTGAGGAGAAACGGTCGG	ATGGTCTGTAGGGCTCGGCC
EpCAM	ACTGGCATCCAAGTGCTTGGTGAT	CGTTGCACTGCTTGGCTTTGAAGA
CK-18	ACACCAACATCACGAGGTTGCA	TGTCCAGTTCCTCACGGTTCTTCT

### Histological evaluation and immunohistochemical analysis

Liver tissues were taken at postoperative day 7 (rejection phase) and day 60 to 85 (tolerogenic phase) from rejecting (DA-LEW) and tolerogenic (DA-PVG) OLT rats, embedded in Tissue-Tek® OCT compound (Sakura Finetek, Torrance, CA, USA), frozen in liquid nitrogen, and stored at −80°C until analysis. Liver tissues from naïve DA rats were used as a control.

For histological evaluation, cryosections (6 µm thick) were fixed with phosphate-buffered paraformaldehyde (4%; pH 7.0) for 10 min at room temperature and stained with hematoxylin and eosin or toluidine blue (Sigma-Aldrich) according to the manufacturer's protocol. All sections were examined using a light microscope (Olympus, Tokyo, Japan).

For immunohistochemical analysis, cryosections (6 µm thick) were fixed with phosphate-buffered paraformaldehyde (4%; pH 7.0) for 10 min at 4°C. Nonspecific proteins were then blocked using Power Block™ Reagent (BioGenex Laboratories, Inc., San Ramon, CA, USA) for 1 hr. The sections were incubated at 4°C for overnight with goat polyclonal Ab against MCP1 (Santa Cruz Biotechnology, Santa Cruz, CA, USA) (×50 dilution with Ab dilution buffer, Dako, Glostrup, Denmark); mouse monoclonal Ab against γδ TCR (×100; BD Biosciences, San Jose, CA, USA), Foxp3 (×200; Santa Cruz Biotechnology), IL-9 (×200; Santa Cruz Biotechnology) or CK-18 (×100; Abcam, Cambridge, MA, USA); rabbit polyclonal Ab against SCF (×200; Santa Cruz Biotechnology), IL-17 (×100; Santa Cruz Biotechnology) or TGF-β1 (×50; Santa Cruz Biotechnology); rabbit monoclonal Ab against EpCAM (×100; Novus Biologicals, Littleton, CO, USA); or rat polyclonal Ab against RT1Ac (×100; AbD Serotec, MorphoSys UK Ltd., Oxford, UK). The sections were rinsed with PBS supplemented with 0.05% Tween-20, then incubated with Alexa Fluor® 488−conjugated donkey anti-goat, mouse or rabbit IgG (×1000; Molecular Probes, Invitrogen Corporation, Carlsbad, CA, USA), HyLite Fluor™ 594−conjugated goat anti-mouse or rabbit IgG (×1000; Ana Spec Inc., Fremont, CA, USA) or Alexa Fluor® 647−conjugated goat anti-rat IgG (×1000; Molecular Probes, Invitrogen Corporation) for 30 min. The nuclei were counterstained with Hoechst 33342 (Molecular Probes, Invitrogen Corporation). All sections were examined using an Olympus FV10i confocal laser scanning microscope.

### SDS-PAGE and Western blot analyses

To detect the protein expression of SCF, IL-9, IL-10, and Foxp3 in livers, naïve DA livers and liver allografts at rejection (DA-PVG and DA-LEW) and during the tolerogenic phase after OLT (DA-PVG) were manually homogenized with T-PER® Tissue Protein Extraction Reagent (Thermo Fisher Scientific Inc., Rockford, IL, USA) supplemented with protease inhibitor complete (Roche Diagnostics, Mannheim, Germany). After centrifugation, liver extracts (100 µg) were run on a 10% sodium dodecyl sulfate-polyacrylamide gel electrophoresis (SDS-PAGE) gel using a mini gel apparatus (Bio-Rad, Burlington, MA, USA), and fractionated proteins were electronically transferred onto a polyvinylidene fluoride transfer membrane (GE Healthcare Bio-Sciences Corp., Piscataway, NJ, USA). The membrane was blocked using 5% skim milk at room temperature for 1 hr and immunoprobed with rabbit polyclonal Ab against SCF (×2000 dilution with 5% skim milk/PBST; Santa Cruz Biotechnology), mouse monoclonal Ab against IL-9 (×2000; Santa Cruz Biotechnology) or Foxp3 (×2000; Santa Cruz Biotechnology) or goat polyclonal Ab against IL-10 (×2000; Santa Cruz Biotechnology) followed by incubation with peroxidase-conjugated goat anti-rabbit IgG (×5000; Cell Signaling, Beverly, MA, USA), goat anti-mouse IgG (×10000; Santa Cruz Biotechnology) or donkey anti-goat IgG (×10000; Santa Cruz Biotechnology). Signals were visualized using an ECL Plus Western Blotting Detection System (GE Healthcare Bio-Sciences Corp.), and relevant bands were quantified by densitometry using a G:BOX Image Station iChemi XL device (Syngene, Cambridge, UK). The data were normalized to actin expression.

### ELISA

To check the hepatic levels of histamine, liver extracts (1 mg) were acylated and assayed in duplicate using a Histamine ELISA kit (Labor Diagnostika Nord GmbH & Co.KG, Nordhorn, Germany). The absorbance at 450 nm was measured using a Victor X4 Multilabel Plate Reader (PerkinElmer, Waltham, MA, USA)

### Preparation of bone marrow-derived mast cells

Bone marrow cells were harvested from femurs of LEW rats and cultured in RPMI 1640 (Sigma-Aldrich, St. Louis, MO, USA) supplemented with 10% FBS, 2 mM L-glutamine, 1 mM sodium pyruvate, 1 mM HEPES, 50 µM 2-mercaptoethanol, 100 U/ml penicillin, and 100 µg/ml streptomycin. The cells were cultured in the presence of 10 ng/ml of recombinant rat IL-3β (PeproTech, Rocky Hill, NJ, USA), and the nonadherent cells were passaged every 3 days. 4 weeks later, the cells were used as mast cells for experiments and referred to as BMMCs [65]. At the time of use, more than 98% of the cells were identified as mast cells by Toluidine blue staining (Fig. 5A).

### Immunophenotyping of splenocytes co-cultured with BMMCs

LEW rat splenocytes were harvested and treated with lysis buffer (150 mM NH_4_Cl, 15 mM NaHCO_3_, 0.1 mM EDTA2Na [pH 7.3]) to lyse red blood cells. After washing twice with PBS, splenocytes were suspended with RPMI-1640 medium (Sigma-Aldrich) supplemented with 10% FBS, 2 mM L-glutamine, 1 mM sodium pyruvate, 1 mM HEPES, 50 µM 2-mercaptoethanol, 100 U/ml penicillin, and 100 µg/ml streptomycin, and splenocytes (2.5×10^6^ cells) were co-cultured with syngeneic BMMCs (0, 1.25, 2.5 and 5×10^6^ cells) and stimulated with Concanavalin A (2.5 µg/ml) at 37°C for 72 hrs in a humidified atmosphere of 5% CO_2_/95% air. For induction of mast cell degranulation, 10 µg/ml of compound 48/80 (Sigma-Aldrich) was added in the culture media. The cells (10^6^ cells/100 µl) were incubated at 4 °C for 30 min with mouse monoclonal Ab against rat TCR γδ chain (1 µg; Santa Cruz Biotechnology), washed twice with PBS and then incubated at 4 °C for 30 min with FITC−conjugated goat anti-mouse Ig (1 µg; BD Biosciences). After washing twice with PBS, the cells were analyzed by LSRII flow cytometer (BD Biosciences).

### Preparation of primary hepatocytes

Primary hepatocytes were purified from naïve LEW rats by the two-step liver perfusion method [66]. Briefly, under anaesthesia by intraperitoneal injection of pentobarbital, LEW rat liver was perfused via the portal vein with buffer I (137 mM NaCl, 5.4 mM KCl, 0.5 mM NaH_2_PO_4_, 0.4 mM Na_2_HPO_4_, 10 mM HEPES, 0.5 mM EGTA, 5 mM D-glucose, pH 7.4) at 37°C, followed by *in situ* digestion with buffer II (137 mM NaCl, 5.4 mM KCl, 5 mM CaCl_2_, 0.5 mM NaH_2_PO_4_, 0.4 mM Na_2_HPO_4_, 10 mM HEPES, 0.05% collagenase IV, pH 7.4) at 37°C. The liver was then excised and hepatocytes were separated from the liver by gently shaking. After filtration, hepatocytes were purified by a percoll gradient centrifugation (GE Healthcare Bio-Sciences Corp.), and washed twice with ice-cold 10% FCS DMEM medium (Invitrogen Corporation). The isolated hepatocytes were suspended in DMEM medium supplemented with 10% FCS, 15 mmol/l HEPES (pH 7.4), 1 µmol/l insulin, 2 mmol/l L-glutamine, 100 units/ml penicillin, and 100 µg/ml streptomycin.

### Cell growth assay

Primary hepatocytes (2×10^5^ cells) were pre-cultured on collagen-coated 6-well plates for 24 hrs, and non-adhesion cells were removed. After addition the new culture medium, hepatocytes were co-cultured with syngeneic BMMCs (0, 2 and 4×10^5^ cells) at 37°C for 72 hrs in a humidified atmosphere of 5% CO_2_/95% air. After washing twice with PBS, new culture medium was added together with MTT reagents (Millipore, Billerica, MA, USA), and cultured for 4 hr. After color development, each sample were transferred to 96-well microtiter plates (Nalge Nunc International, Roskilde, Denmark), and the absorbance (595 nm) was measured using a Victor X4 Multilabel Plate Reader (PerkinElmer).

### Statistical analysis

Student's *t*-tests were used to determine the significance of the difference between normally distributed means of value in two groups. Each sample was tested in triplicate, and results are indicated as mean ± SD.

## References

[1] Starzl TE, Marchioro TL, Vonkaulla KN, Hermann G, Brittain RS (1963). Homotransplantation of the Liver in Humans.. Surg Gynecol Obstet.

[2] Calne RY, Rolles K, White DJ, Thiru S, Evans DB (1979). Cyclosporin A initially as the only immunosuppressant in 34 recipients of cadaveric organs: 32 kidneys, 2 pancreases, and 2 livers.. Lancet.

[3] Chu Z, Zhang J, Zhao Y, Ji Q, Zhong J (2010). Influence of immunosuppressive drugs on the development of CD4(+)CD25(high) Foxp3(+) T cells in liver transplant recipients.. Transplant Proc.

[4] van de Wetering J, Koumoutsakos P, Peeters A, van der Mast BJ, de Kuiper P (2011). Discontinuation of calcineurin inhibitors treatment allows the development of FOXP3+ regulatory T-cells in patients after kidney transplantation.. Clin Transplant.

[5] Shen Z, Song Q, Chen L, Zhong B, Tang S (2011). Bidirectional immunoregulation of calcineurin inhibitor tacrolimus on FOXP3 transcription?. Med Hypotheses.

[6] Denecke C, Reutzel-Selke A, Sawitzki B, Boenisch O, Khalepy Z (2012). Low-dose cyclosporine mediates donor hyporesponsiveness in a fully mismatched rat kidney transplant model..

[7] Koshiba T, Li Y, Takemura M, Wu Y, Sakaguchi S (2007). Clinical, immunological, and pathological aspects of operational tolerance after pediatric living-donor liver transplantation.. Transpl Immunol.

[8] Martinez-Llordella M, Puig-Pey I, Orlando G, Ramoni M, Tisone G (2007). Multiparameter immune profiling of operational tolerance in liver transplantation.. Am J Transplant.

[9] Demetris AJ (1995). Terminology for hepatic allograft rejection. International Working Party.. Hepatology.

[10] Batts KP (1999). Acute and chronic hepatic allograft rejection: pathology and classification.. Liver Transpl Surg.

[11] O'Keeffe C, Baird AW, Nolan N, McCormick PA (2002). Mast cell hyperplasia in chronic rejection after liver transplantation.. Liver Transpl.

[12] Matsunaga Y, Kawasaki H, Terada T (1999). Stromal mast cells and nerve fibers in various chronic liver diseases: relevance to hepatic fibrosis.. Am J Gastroenterol.

[13] Farrell DJ, Hines JE, Walls AF, Kelly PJ, Bennett MK (1995). Intrahepatic mast cells in chronic liver diseases.. Hepatology.

[14] Armbrust T, Batusic D, Ringe B, Ramadori G (1997). Mast cells distribution in human liver disease and experimental rat liver fibrosis. Indications for mast cell participation in development of liver fibrosis.. J Hepatol.

[15] Gaca MD, Pickering JA, Arthur MJ, Benyon RC (1999). Human and rat hepatic stellate cells produce stem cell factor: a possible mechanism for mast cell recruitment in liver fibrosis.. J Hepatol.

[16] Yamashiro M, Kouda W, Kono N, Tsuneyama K, Matsui O (1998). Distribution of intrahepatic mast cells in various hepatobiliary disorders. An immunohistochemical study.. Virchows Arch.

[17] Khalil RM, Luz A, Mailhammer R, Moeller J, Mohamed AA (1996). Schistosoma mansoni infection in mice augments the capacity for interleukin 3 (IL-3) and IL-9 production and concurrently enlarges progenitor pools for mast cells and granulocytes-macrophages.. Infect Immun.

[18] Lajoie G, Nadasdy T, Laszik Z, Blick KE, Silva FG (1996). Mast cells in acute cellular rejection of human renal allografts.. Mod Pathol.

[19] Abo-Zenah H, Katsoudas S, Wild G, de Takats D, Shortland J (2002). Early human renal allograft fibrosis: cellular mediators.. Nephron.

[20] Goto E, Honjo S, Yamashita H, Shomori K, Adachi H (2002). Mast cells in human allografted kidney: correlation with interstitial fibrosis.. Clin Transplant.

[21] Yousem SA (1997). The potential role of mast cells in lung allograft rejection.. Hum Pathol.

[22] Li QY, Raza-Ahmad A, MacAulay MA, Lalonde LD, Rowden G (1992). The relationship of mast cells and their secreted products to the volume of fibrosis in posttransplant hearts.. Transplantation.

[23] Koskinen PK, Kovanen PT, Lindstedt KA, Lemstrom KB (2001). Mast cells in acute and chronic rejection of rat cardiac allografts – a major source of basic fibroblast growth factor.. Transplantation.

[24] Lu LF, Lind EF, Gondek DC, Bennett KA, Gleeson MW (2006). Mast cells are essential intermediaries in regulatory T-cell tolerance.. Nature.

[25] Boerma M, Fiser WP, Hoyt G, Berry GJ, Joseph L (2007). Influence of mast cells on outcome after heterotopic cardiac transplantation in rats.. Transpl Int.

[26] Kamada N, Davies HS, Roser B (1981). Reversal of transplantation immunity by liver grafting.. Nature.

[27] Kamada N, Wight DG (1984). Antigen-specific immunosuppression induced by liver transplantation in the rat.. Transplantation.

[28] Kamada N (1985). Transfer of specific immunosuppression of graft rejection using lymph from tolerant liver-grafted rats.. Immunology.

[29] Lord R, Kamada N, Kobayashi E, Goto S, Sunagawa M (1995). Isolation of a 40 kDa immunoinhibitory protein induced by rat liver transplantation.. Transpl Immunol.

[30] Andrzejewski W, Brolsch C (1982). Postoperative reactions of rats after orthotopic liver transplantation: a model for the human response? A histological and biochemical study.. Eur Surg Res.

[31] Bearman SI (1997). Use of stem cell factor to mobilize hematopoietic progenitors.. Curr Opin Hematol.

[32] Zhang W, Wu K, He W, Gao Y, Huang W (2010). Transforming growth factor beta 1 plays an important role in inducing CD4(+)CD25(+)forhead box P3(+) regulatory T cells by mast cells.. Clin Exp Immunol.

[33] Roark CL, Simonian PL, Fontenot AP, Born WK, O'Brien RL (2008). gammadelta T cells: an important source of IL-17.. Curr Opin Immunol.

[34] O'Brien RL, Roark CL, Born WK (2009). IL-17-producing gammadelta T cells.. Eur J Immunol.

[35] Do JS, Fink PJ, Li L, Spolski R, Robinson J (2010). Cutting edge: spontaneous development of IL-17-producing gamma delta T cells in the thymus occurs via a TGF-beta 1-dependent mechanism.. J Immunol.

[36] Paton WD (1951). Compound 48/80: a potent histamine liberator.. Br J Pharmacol Chemother.

[37] Zaruba MM, Theiss HD, Vallaster M, Mehl U, Brunner S (2009). Synergy between CD26/DPP-IV inhibition and G-CSF improves cardiac function after acute myocardial infarction.. Cell Stem Cell.

[38] Yovchev MI, Grozdanov PN, Joseph B, Gupta S, Dabeva MD (2007). Novel hepatic progenitor cell surface markers in the adult rat liver.. Hepatology.

[39] Yoon SM, Gerasimidou D, Kuwahara R, Hytiroglou P, Yoo JE (2011). Epithelial cell adhesion molecule (EpCAM) marks hepatocytes newly derived from stem/progenitor cells in humans.. Hepatology.

[40] Fiegel HC, Park JJ, Lioznov MV, Martin A, Jaeschke-Melli S (2003). Characterization of cell types during rat liver development.. Hepatology.

[41] Gassel HJ, Engemann R, Thiede A, Hamelmann H (1987). Replacement of donor Kupffer cells by recipient cells after orthotopic rat liver transplantation.. Transplant Proc.

[42] Gouw AS, Houthoff HJ, Huitema S, Beelen JM, Gips CH (1987). Expression of major histocompatibility complex antigens and replacement of donor cells by recipient ones in human liver grafts.. Transplantation.

[43] Petersen BE, Bowen WC, Patrene KD, Mars WM, Sullivan AK (1999). Bone marrow as a potential source of hepatic oval cells.. Science.

[44] Theise ND, Nimmakayalu M, Gardner R, Illei PB, Morgan G (2000). Liver from bone marrow in humans.. Hepatology.

[45] Theise ND, Badve S, Saxena R, Henegariu O, Sell S (2000). Derivation of hepatocytes from bone marrow cells in mice after radiation-induced myeloablation.. Hepatology.

[46] Wu T, Cieply K, Nalesnik MA, Randhawa PS, Sonzogni A (2003). Minimal evidence of transdifferentiation from recipient bone marrow to parenchymal cells in regenerating and long-surviving human allografts.. Am J Transplant.

[47] Romagnani S (2006). Regulation of the T cell response.. Clin Exp Allergy.

[48] Cao Y, Zhao J, Lei Z, Shen S, Liu C (2008). Local accumulation of FOXP3+ regulatory T cells: evidence for an immune evasion mechanism in patients with large condylomata acuminata.. J Immunol.

[49] Li J, Lai X, Liao W, He Y, Liu Y (2011). The dynamic changes of Th17/Treg cytokines in rat liver transplant rejection and tolerance.. Int Immunopharmacol.

[50] Girardi M (2006). Immunosurveillance and immunoregulation by gammadelta T cells.. J Invest Dermatol.

[51] Nakano T, Kawamoto S, Lai CY, Sasaki T, Aki T (2004). Liver transplantation-induced antihistone H1 autoantibodies suppress mixed lymphocyte reaction.. Transplantation.

[52] Nakano T, Goto S, Lai CY, Hsu LW, Kao YH (2007). Experimental and clinical significance of antinuclear antibodies in liver transplantation.. Transplantation.

[53] Nakano T, Goto S, Lai CY, Hsu LW, Ono K (2007). Impact of vaccine therapy using nuclear histone H1 on allograft survival in experimental organ transplantation.. Transpl Immunol.

[54] Nakano T, Goto S, Lai CY, Hsu LW, Wong JL (2008). Involvement of autoimmunity against nuclear histone H1 in liver transplantation tolerance.. Transpl Immunol.

[55] Parseghian MH, Luhrs KA (2006). Beyond the walls of the nucleus: the role of histones in cellular signaling and innate immunity.. Biochem Cell Biol.

[56] Lotze MT, Tracey KJ (2005). High-mobility group box 1 protein (HMGB1): nuclear weapon in the immune arsenal.. Nat Rev Immunol.

[57] Hershko AY, Suzuki R, Charles N, Alvarez-Errico D, Sargent JL (2011). Mast cell interleukin-2 production contributes to suppression of chronic allergic dermatitis.. Immunity.

[58] Yang Z, Zhang B, Li D, Lv M, Huang C (2010). Mast cells mobilize myeloid-derived suppressor cells and Treg cells in tumor microenvironment via IL-17 pathway in murine hepatocarcinoma model.. PLoS One.

[59] Truta-Feles K, Lagadari M, Lehmann K, Berod L, Cubillos S (2010). Histamine modulates gammadelta-T lymphocyte migration and cytotoxicity, via Gi and Gs protein-coupled signalling pathways.. Br J Pharmacol.

[60] Ren X, Hogaboam C, Carpenter A, Colletti L (2003). Stem cell factor restores hepatocyte proliferation in IL-6 knockout mice following 70% hepatectomy.. J Clin Invest.

[61] Hu B, Colletti LM (2008). Stem cell factor and c-kit are involved in hepatic recovery after acetaminophen-induced liver injury in mice.. Am J Physiol Gastrointest Liver Physiol.

[62] Ren X, Hu B, Colletti L (2008). Stem cell factor and its receptor, c-kit, are important for hepatocyte proliferation in wild-type and tumor necrosis factor receptor-1 knockout mice after 70% hepatectomy.. Surgery.

[63] Michalopoulos GK (2007). Liver regeneration.. J Cell Physiol.

[64] Kamada N, Calne RY (1979). Orthotopic liver transplantation in the rat. Technique using cuff for portal vein anastomosis and biliary drainage.. Transplantation.

[65] Huang B, Lei Z, Zhang GM, Li D, Song C (2008). SCF-mediated mast cell infiltration and activation exacerbate the inflammation and immunosuppression in tumor microenvironment.. Blood.

[66] Tateno C, Takai-Kajihara K, Yamasaki C, Sato H, Yoshizato K (2000). Heterogeneity of growth potential of adult rat hepatocytes in vitro.. Hepatology.

